# The Combination of Selenium and LED Light Quality Affects Growth and Nutritional Properties of Broccoli Sprouts

**DOI:** 10.3390/molecules25204788

**Published:** 2020-10-19

**Authors:** Rui He, Meifang Gao, Rui Shi, Shiwei Song, Yiting Zhang, Wei Su, Houcheng Liu

**Affiliations:** College of Horticulture, South China Agricultural University, Guangzhou 510642, China; ruihe@stu.scau.edu.cn (R.H.); gmf@stu.scau.edu.cn (M.G.); ruishi@stu.scau.edu.cn (R.S.); swsong@scau.edu.cn (S.S.); yitingzhang@scau.edu.cn (Y.Z.); susan_l@scau.edu.cn (W.S.)

**Keywords:** broccoli sprouts, selenium, light quality, growth, health-maintaining compounds

## Abstract

Selenium (Se) supplement was combined with different LED light qualities to investigate mutual effects on the growth, nutritional quality, contents of glucosinolates and mineral elements in broccoli sprouts. There were five treatments: CK:1R1B1G, 1R1B1G+Se (100 μmol L^−1^ Na_2_SeO_3_), 1R1B+Se, 1R2B+Se, 2R1B+Se, 60 μmol m^−2^ s^−1^ PPFD, 12 h/12 h (light/dark). Sprouts under a combination of selenium and LED light quality treatment exhibited no remarkable change fresh weight, but had a shorter hypocotyl length, lower moisture content and heavier dry weight, especially with 1R2B+Se treatment. The contents of carotenoid, soluble protein, soluble sugar, vitamin C, total flavonoids, total polyphenol and contents of total glucosinolates and organic Se were dramatically improved through the combination of Se and LED light quality. Moreover, heat map and principal component analysis showed that broccoli sprouts under 1R2B+Se treatment had higher nutritional quality and health-promoting compound contents than other treatments. This suggests that the Se supplement under suitable LED lights might be beneficial to selenium-biofortified broccoli sprout production.

## 1. Introduction

Broccoli (*Brassica oleracea* L. var. *italica*) has received considerable attention for its rich bioactive composition, especially with respect to glucosinolates (GLs), which can reduce the risk of different diseases, including cancer, inflammation, cardiovascular and neurodegenerative diseases [1]. In recent years, the consumption of broccoli sprouts has increased, along with consumer awareness and appreciation for their diverse flavors, vivid colors, delicate textures and phytonutrients, including GLs.

Selenium is an essential micro-nutrient that plays critical roles in various physiological processes, such as thyroid hormone metabolism, antioxidant defenses and immune function [2]. Additionally, it plays multiple roles in growth and function of living cells and has many crucial biological functions in animals and humans [3]. The development and uses of Se-biofortified agricultural products have been proposed as a promising functional agricultural strategy to increase selenium supplementation and dietary nutrient intake for humans [4]. Selenium has a significant impact on growth and quality of vegetables. Addition of selenium significantly increases Se content in spinach [5], broccoli [6], cabbages [7], radishes [8], generally without negatively affecting the biomass and quality of plants. Selenium promotes metabolic activities, particularly antioxidant activities, enhancing plant tolerance under unfavorable growth conditions [9]. Selenium addition increases glucosinolate production in radish roots [8], and influences the expression of genes involved in the biosynthesis and degradation of glucosinolates in Arabidopsis [10]. Brassica can accumulate high Se concentrations through its metabolic capacity to convert selenoamino acids into non-proteinogenic amino acids, such as selenomethyl selenocysteine (SeMSC), c-glutamyl SeMSC and selenocystathionine [11]. Thus, selenium enrichment of sprouts can enhance the anticancer properties of broccoli sprouts due to the accumulation of two active anticancer agents: sulforaphane and Se-methylselenocysteine. An amount of 100 μmol L^−1^ selenate and selenite treatments have been shown to significantly affect the contents of health-promoting compounds in broccoli sprouts from different cultivars [12]. Once absorbed by plants, selenite remains in organic form and becomes a more effective inducer of glutathione peroxidase [13].

Blue (B) and red (R) light-emitting diodes (LEDs) are commonly used for plant growth in view of their roles as essential energy sources for photosynthetic carbon assimilation [14]. Combined R and B LEDs are regarded as the optimal light quality to support the growth of most plants. Red plus blue light is beneficial to cucumber seedlings’ development, and plant height, dry matter quality and Fv/Fm are notably increased compared with monochromatic R or B light treatment [15]. Bigger leaves and heavier fresh weight were found in red leaf lettuce when seedlings were grown under mixed R and B light in comparison to those grown under monochromatic R light [16]. B light supplemented with R light positively affected plant biomass in nine tomato genotypes and led to the increased concentration of soluble protein, chlorophyll and carotenoid [17].

Primary and secondary metabolites in specialty vegetable crops will be higher under narrow-band LED lights, due to plants only receiving B and R wavelengths compared with other wavelengths found in the spectra emitted from fluorescent/incandescent bulbs. It is possible to reduce possible light stress that plants are subjected to in the growing environment by using narrow-band wavelengths [18]. Stem elongation, stomatal opening, and anthocyanin accumulation were inhibited under green light due to plant responses to green light tending to neutralize blue- or red-induced responses [19]. Green (G) light led to a bigger leaf length and width of ‘Rex’ and ‘Rouxai’ lettuce and ‘Siberian’ kale plants, as well as shoot fresh weight in ‘Siberian’ kale plants, compared with combined R and B light [20]. Biomass and leaf area could be further increased in lettuce with the addition of G light to R and B LEDs [21]. It is possible that the combination of Se and LED light quality may be beneficial to the production of selenium-biofortified high-quality broccoli sprouts. Thus, this study investigates the combination of selenium and different ratios of B/R/G narrow-band LED light quality on growth, biomass, physicochemical properties and mineral elements in broccoli sprouts in an artificial light plant factory.

## 2. Results 

### 2.1. Growth and Weight

Selenium supplementation combined with different LED light qualitites had a significant impact on the growth of broccoli sprouts (Table 1). Sprouts under 1R1B1G+Se, 1R1B+Se, 1R2B+Se and 2R1B+Se had significantly shorter hypocotyl lengths than under 1R1B1G. Sprouts’ fresh weight did not differ among the treatments. Dry weight under all Se-treated sprouts was considerably greater than under 1R1B1G, and the heaviest sprout dry weight occurred under 1R2B+Se. Moisture content was dramatically decreased with all Se treatments, but there was no significant difference among them.

### 2.2. Pigment Content in Broccoli Sprouts

A combination of Se and LED light quality treatment significantly affected broccoli sprouts’ pigment content (Table 2). The content changes of chlorophyll a, chlorophyll b, total chlorophyll were consistent among all treatments. Chlorophyll content of Se-treated sprouts was significantly reduced compared with 1R1B1G, but no significant differences were found with 1R2B+Se. The carotenoid contents under 1R1B1G+Se, 1R1B+Se and 2R1B+Se notably increased, by 15.79%, 21.05% and 21.05%, respectively, compared to 1R1B1G. Similar to carotenoid contents, total anthocyanin contents were significantly higher under all combinations of Se and LED light treatment when compared with 1R1B1G. The ratios of Chl a/b did not differ among the treatments, while the Chl/Car ratios under 1R1B1G+Se, 1R1B+Se and 2R1B+Se were significantly lower than under 1R1B1G and 1R2B+Se.

### 2.3. The Content of Soluble Sugars, Soluble Protein in Broccoli Sprouts

**Figure 1:**
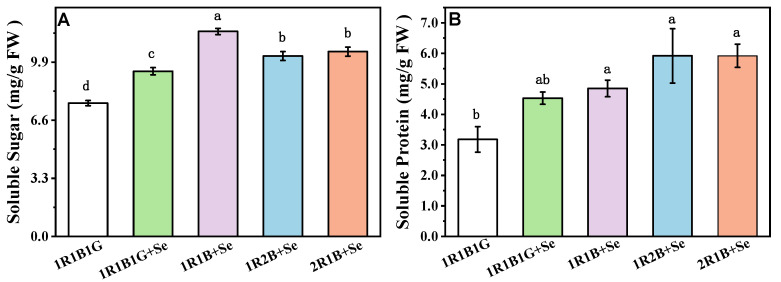
The contents of soluble sugars (**A**) and soluble protein (**B**) of broccoli sprouts grown under a combination of selenium and LED light quality treatments. The letters marked in all figures indicate the significance of the difference (*p* ≤ 0.05, Duncan’s test).

As shown in Figure 1, the contents of soluble sugar and soluble protein in broccoli sprouts were significantly increased by the combination of Se and LED light quality treatment. The highest soluble sugar content was observed with 1R1B+Se, with an increase of 53.90%, and 1R1B1G +Se, 1R2B+Se, and 2R1B+Se resulted in an increase of 23.91%, 35.40%, and 38.70%, respectively (Figure 1A). Compared with 1R1B1G, the soluble protein contents with 1R1B1G+Se, 1R1B+Se, 1R2B+Se, and 2R1B+Se were significantly higher, by 42.45%, 52.52%, 86.11% and 86.16%, respectively (Figure 1B).

### 2.4. The Contents of Vitamin C (Vc), Total Phenolic Compounds (TPC) and Total Flavoncids (TF) in Broccoli Sprouts

**Figure 2:**
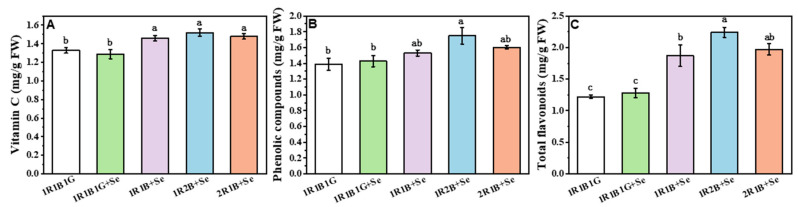
The content of Vc (**A**), TPC (**B**) and TF (**C**) of broccoli sprouts grown under a combination of selenium and LED light quality treatments. The letters marked in all figures indicate the significance of the difference (*p* ≤ 0.05, Duncan’s test).

The contents of Vc, TPC, and TF in broccoli sprouts were affected by the combination of Se and LED light quality treatment (Figure 2). Compared with 1R1B1G, the Vc content of broccoli sprouts was not affected by 1R1B1G+Se, while those with 1R1B+Se, 1R2B+Se and 2R1B+Se were significantly higher than 1R1B1G, by 9.77%, 14.29% and 11.28%, respectively (Figure 2A). The TPC contents with 1R1B+Se, 1R2B+Se and 2R1B+Se were higher than with 1R1B1G, by 10.07%, 25.9%, and 15.11%, respectively, but no significant differences were found between 1R1B1G +Se and 1R1B1G (Figure 2B). Similar to TPC content, higher TF contents were observed with 1R1B+Se, 1R2B+Se and 2R1B+Se, and these were significantly higher than 1R1B1G, by 53.28%, 83.61% and 61.48%, respectively (Figure 2C).

### 2.5. The Mineral Element Content in Broccoli Sprouts

Selenium supplementation combined with different LED light quality treatments significantly affected the contents of organic Se, K, Ca, Mg, S and Zn in broccoli sprouts (Table 3). The highest organic Se contents of sprouts were found with 1R1B+Se and 1R2B+Se, followed by 2R1B+Se and 1R1B1G+Se. The S content in 1R1B1G+Se was higher than 1R1B1G, and the highest S content was found in 1R1B+Se. However, the contents of K, Ca and Zn in broccoli sprouts were significantly lower under all Se treatments in comparison to 1R1B1G. The contents of N, P, Fe were not significantly affected by the combination of Se and LED light quality treatments.

### 2.6. The Glucosinolate (GL) Contents in Broccoli Sprouts

**Figure 3:**
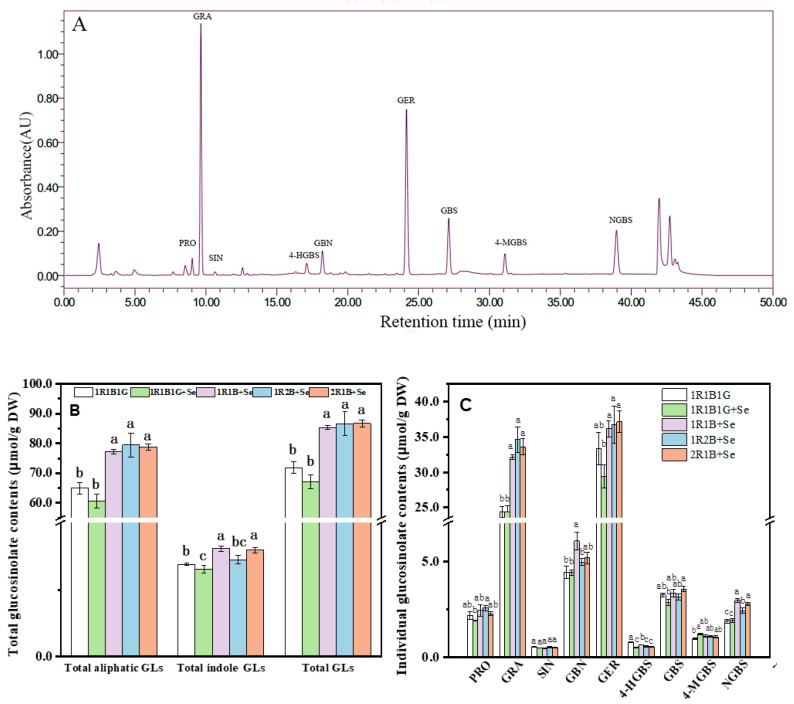
High-Performance Liquid Chromatography of identified glucosinoaltes from broccoli sprouts (**A**) the total glucosinolate contents (**B**) and the individual glucosinolate contents (**C**) in broccoli sprouts. The letters marked in all figures indicate the significance of the difference (*p* ≤ 0.05, Duncan’s test).

Nine GLs were identified by HPLC in broccoli sprouts (Figure 3), which comprised five aliphatic GLs (progoitrin (PRO), glucoraphanin (GRA), sinigrin (SIN), glucobrassicanapin (GBN) and glucoerucin (GRE)), and four indole GLs (4-hydroxy-glucobrassicin (4-HGBS), glucobrassicin (GBS), 4-methoxy-glucobrassicin (4-MGBS) and neoglucobrassicin (NGBS)). Aliphatic GL content accounted for approximately 70% of the total GL content in broccoli sprouts in this study. With a noticeable decrease in the content of PRO, GER, 4HGBS and GBS, the contents of total aliphatic GLs and total GLs in broccoli sprouts decreased with 1R1B1G+Se treatment compared to with 1R1B1G. The increase in total GL content was mainly due to the increase of aliphatic GL contents, which increased by 18.95%, 22.31% and 21.25% in 1R1B+Se, 1R2B+Se and 2R1B+Se, respectively, but there was no significant difference among these treatments. GRA and GER, as two major aliphatic GLs were not affected by 1R1B1G+Se, while they were significantly increased by 1R1B+Se, 1R2B+Se and 2R1B+Se. 4-HGBS content decreased under a combination of Se and LED light quality treatment, while 4-MGBS and NGBS contents increased under these treatments.

### 2.7. Heat Map Analysis

**Figure 4:**
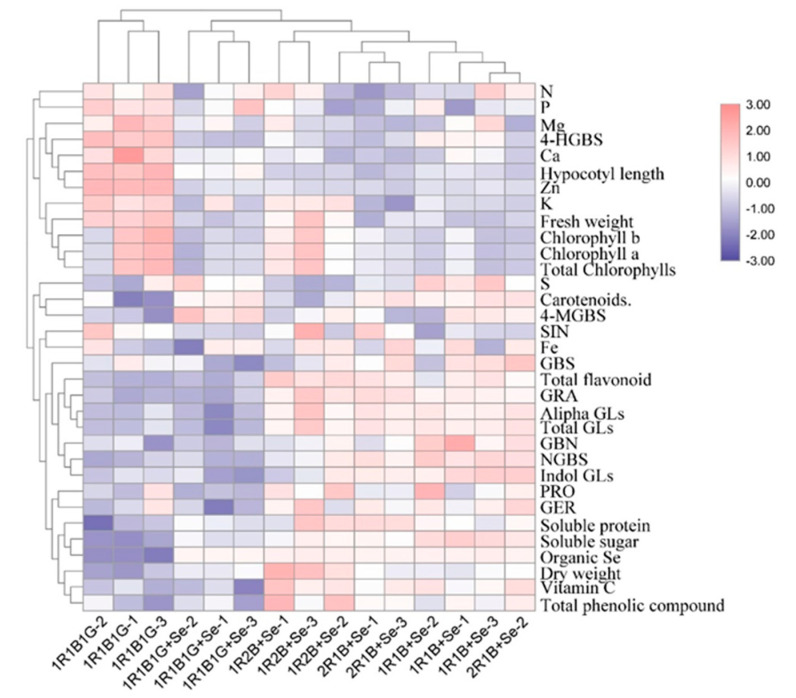
Cluster heat map analysis of broccoli sprouts grown under a combination of selenium and LED light quality treatments. Results are visualized using a false color scale, with blue indicating a decrease and pale red indicating an increase of the response parameters.

A heat map was used to analyze the response among the investigated parameters and showed a broad view of the effects of the combination of selenium and LED light quality on broccoli sprouts (Figure 4). The 1R1B+Se, 1R2B+Se and 2R1B+Se clusters, which are marked close to each other in the heat map, indicate the similarity of their impacts, while they are equidistant from the 1R1B1G and 1R1B1G+Se clusters. 1R1B1G was characterized by a higher fresh weight, hypocotyl length, content of chlorophyll and N, P, K, Ca, Mg in the broccoli sprouts, which contributed to separating the 1R1B1G cluster from the others; 1R1B+Se, 1R2B+Se, and 2R1B+Se treatments had higher contents of antioxidants, glucosinolates and organic Se content.

### 2.8. Principal Component Analysis

**Figure 5:**
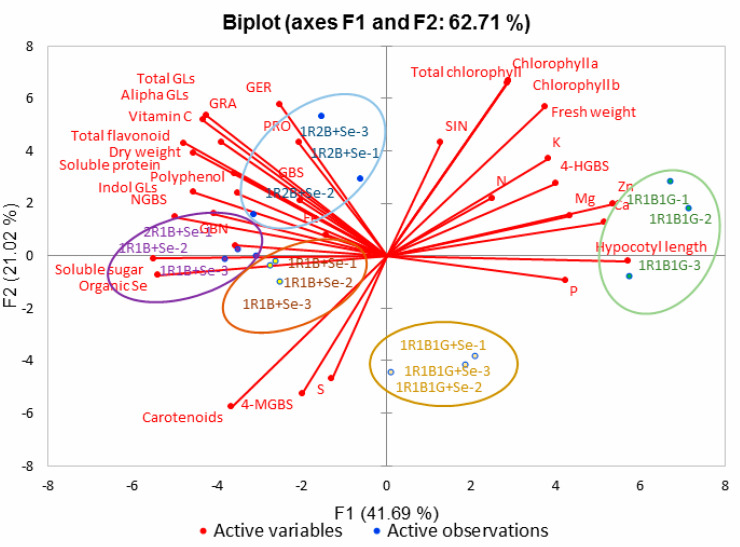
Principal component analysis of broccoli sprouts grown under a combination of selenium and LED light quality treatments.

The most significant overall effects of the combination of selenium and LED light quality on growth, phytochemical composition and mineral element of broccoli sprouts were assessed by principal component analysis (PCA) (Figure 5). The computed model captured 62.71% of the total observed variance with the first two principal components (PCs). Score and loading plots on PC2 vs. PC1 are shown in Figure 5. Combination of selenium and LED light quality had distinct impacts on growth, primary and secondary metabolites, and mineral elements in broccoli sprouts. PCA and heat mapping showed similar results: there were significant differences between 1R1B1G, 1R1B1G+Se, 1R1B+Se and 1R2B+Se, according to the PCA scatter plot (Figure 5). The PCA correlation circle (Figure 5) indicates that fresh weight and chlorophyll contents were positively correlated with N, K, Mg contents. Additionally, glucosinolate contents were strongly correlated with the contents of Vc, TPC and TF in broccoli sprouts.

## 3. Discussion 

### 3.1. Effects of Combination of Selenium and LED Light Quality on Broccoli Sprout Growth

The combination of selenium and LED light quality had a negative effect on the growth of broccoli sprouts, resulting in a reduction in hypocotyl elongation and fresh weight, as well as moisture content (Table 1). Low Se concentrations enhance plant growth, while high Se concentrations are extremely toxic to organisms. Selenium can replace sulfur in proteins and negatively affect growth and germination of seeds and reduce yields [22,23]. Different B/R light ratios play virtual roles in plant growth and pigment accumulation. When cucumber seedlings were cultivated under various R-to-B light ratios (R:B = 2.5:7.5, 5:5, 7:3, and 9:1) for 17 days, plant height, hypocotyl and epicotyl length, leaf area, fresh and dry mass decreased as the B light ratio decreased [24]. In this study, although shorter hypocotyl length was found in Se-treated broccoli sprouts, the combination of selenium and LED light quality did not affect the fresh weight, and increased the dry weight of broccoli sprouts. Higher B/R light (1R2B+Se) led to heavier fresh and dry weight than other Se-treated broccoli sprouts (Table 1). This indicates that an appropriate concentration of Se supplementation under suitable LED lights could significantly increase the growth and biomass of broccoli sprouts.

### 3.2. Effects of Combination of Selenium and LED Light Quality on Pigment Content in Broccoli Sprouts

Previous studies have shown that a significant increase in chlorophyll content (chlorophyll a, chlorophyll b and total chlorophyll) can be observed in lettuce [25] and broccoli [26] under Se treatments. Selenium applications (60 and 120 mg·L^−1^) reduced chlorophyll content in basil, leading to higher contents of carotenoid [27]. In this study, the addition of Se significantly reduced the chlorophyll content in broccoli sprouts, while remarkably increasing the content of carotenoids and anthocyanin (Table 2). An increase in the carotenoid contents of Se-treated sprouts may be caused by the mitigation of photoinhibition on the photosynthetic apparatus, mainly via the xanthophyll cycle [28]. Many plant species are more sensitive to R light and B light than to other light wavelengths, as chlorophyll a and b efficiently absorb B and R light [29]. In this study, higher concentrations of pigments responded to a higher portion of B light in the Se-treated sprouts (1R2B+Se) (Table 2). The enhanced content of chlorophyll and carotenoid and anthocyanin in broccoli sprouts under higher B/R light treatment might be due to the cryptochrome action under high B irradiance [30].

### 3.3. Effects of Combination of Selenium and LED Light Quality on Phytochemical Content in Broccoli Sprouts

Soluble sugars and soluble proteins are important parameters in evaluating the nutritional quality of vegetables, because they provide essential proteins and energy for the human body. Selenium treatment plays a pivotal role in increasing quality parameters of tomato fruit, such as soluble solid content [31], contents of glucose, fructose, and total sugar [32]. This study showed that the content of soluble sugars and soluble proteins in broccoli sprouts was significantly increased by Se treatment (Figure 1), especially under 1R1B+Se, 1R2B+Se, and 2R1B+Se. Soluble sugar contents were higher in tomato seedlings under B light than under other LED light, whereas the highest amount of soluble proteins was observed under a mixture of R and B LED light [33]. Blue LED light or a mixture of R and B LED light led to a higher content of soluble proteins in radish seedlings compared with white or R LED light [34]. Compared to other light wavelengths, R and B light are more effective for increasing contents of soluble sugar and protein in vegetables, possibly because R and B light are the two major lights driving photosynthate biosynthesis, and B light facilitates the protein biosynthesis, while preventing its degeneration [35]. Thus, the combination of Se supplement under the higher B: R light treatment might be beneficial to improve nutrition quality in broccoli sprouts.

Vitamin C (Vc) provides vital antioxidants in plants, and plays a significant role in plant stress response and cell wall metabolism and expansion [36]. Vitamin C contents in broccoli sprouts significantly increased under 1R1B+Se, 1R2B+Se, and 2R1B+Se, whereas no significant difference was observed between 1R1B1G+Se and 1R1B1G, indicating that Se supplementation had no significant effect on Vc contents in broccoli sprouts (Figure 2). Blue LED light and a mixture of R and B LED light resulted in higher Vc contents in lettuce than R LED light [37]. Irradiation from blue fluorescent lamps led to higher Vc concentrations in lettuce and komatsuna, partly due to the fact that B light could increase the plant photosynthesis capacity and increase the synthesis and accumulation of hexose and D-glucose, which are Vc precursors and can stimulate Vc synthesis via several metabolic pathways [38]. Hence, the combination of Se supplement and a higher B/R light ratios LED lights appears to be more effectively facilitating Vc biosynthesis and accumulation in broccoli sprouts.

Phytochemicals, including anthocyanins, polyphenols, and flavonoids, play an important role in the protection of cardiovascular health and prevention of certain cancers [39]. A combination of Se supplementation under 1R1B1G light did not cause higher content of TPC and TF in broccoli sprouts, but anthocyanin content in broccoli sprouts was significantly increased by the combination of Se supplementation and LED light quality. In basil, Se did not induce changes in phenolic compound content [40], but it might positively affect the anthocyanin content. The addition of B light to R light or increased B light intensity increased total phenolic concentration and the antioxidant capacity of red leaf lettuce [41]. Similarly, the concentration of total phenolic and total flavonoid, and antioxidant capacity of lettuces grown under high ratios of B LED (such as B:R = 35:65, 47:53 or 59:41) were significantly higher compared with the treatments of B:R = 0:100, 13:87 and 26:74 [42]. In this study, although 1R1B1G+Se did not cause higher content of TPC and TF in broccoli sprouts, the anthocyanin content in broccoli sprouts was significantly increased by the combination of Se supplement and LED light quality. The TPC and TF content of broccoli sprouts significantly increased in 1R1B+Se, 1R2B+Se, and 2R1B+Se, and the highest content was observed in 1R2B+Se (Figure 3). The concentrations of phenolics and flavonoids in green and purple basil plants were both decreased by G light radiation [43]. It was postulated that the decreased phytochemical concentration in green light treatments was caused by the co-actions of decreased R and/or B light proportions and an increasing reversal of B light-induced effects by G light [43]. On the other hand, B light could activate the expression of *PAL* (phenylalanine ammonia-lyase), *CHS* (chalcone synthesis), and *DFR* (dihydroflavono l-4-reductase), which are key elements in the biosynthetic pathways of anthocyanin and flavonoid compounds [44]. Thus, Se supplementation under higher B/R light ratios LED lights might be strategically employed to enhance many bioactive compounds and consequently the nutrition of vegetables.

### 3.4. Effects of Combination of Selenium and LED Light Quality on Mineral Element Content in Broccoli Sprouts

Mineral nutrients play a crucial role in plant physiology and metabolism. Organic selenium is more bio-available to the human body than inorganic selenium [45]. The content of organic Se in broccoli sprouts remarkably increased under the combination of Se supplement and LED light, but the contents of K, Ca and Zn decreased (Table 3). Meanwhile, S content in broccoli sprouts increased under 1R1B1G+Se compared to 1R1B1G, indicating that Se supplementation could promote the accumulation of S. When spraying high-concentration Se (100 μg·mL^−1^), more S may be required for appropriate translocation, and thus broccoli takes up more S to meet this demand [26]. The contents of N, P and K in broccoli heads were not affected by Se spray at 10, 50, and 100 μg·mL^−1^ [26]. This might indicate that the effects of Se on mineral nutrients were dependent on the species and concentration. The concentrations of Ca, Mg, Mn and Fe were higher in lettuce grown under B or R + B LEDs compared to under other light [46]. The accumulation of Fe, P, Mg, and N in lettuce tissues was significantly increased by either R or a mixture of R and B (R:B = 2), while the accumulation of K and Ca increased under B light [47]. However, no differences in N accumulation in lettuce leaves were found among monochromatic B or R lights or mixtures of R and B at varying ratios (e.g., R:B = 0.5, 1, 3, and 13) [48]. In general, B light could affect proton pumping, membrane permeability, and ion channel activities in plants [49]. Hence, Se supplementation under higher ratios of B and R LED light might be an optimal and efficient method for the production of organic Se in broccoli sprouts.

### 3.5. Effects of Combination of Selenium and LED Light Quality on Glucosinolates Contents in Broccoli Sprouts

Glucosinolates, which contain nitrogen and sulfur in their structures, are the major class of secondary metabolites found in Brassica crops, and play important roles in human body nutrition and health [50]. Selenium biofortification and light quality can affect GL production in Brassica. 1R1B1G+Se significantly reduced the content of total aliphatic GLs and total GLs, but significantly increased the content of 4-MGBS in broccoli sprouts (Figure 4). Selenium application reduced total GL content in radish leaves, causing a 35% increase in radish roots [8]. Selenium application could alter GL contents in broccoli due to the fact that Se treatment significantly down-regulated D-glyceric acid, succinic acid and citric acid, serine O-acetyltransferase [51]. Meanwhile, Se treatment could influence β-alanine metabolism, glutathione metabolism, and biosynthesis of plant secondary metabolites in broccoli sprouts [51]. The repression of genes expression, such as *BCAT4*, *MAM1*, *CYP79B2* and *FMO2*, were the cause of inhibition of glucosinolate accumulation in the Se-treated broccoli [52]. In addition, *MYB28* and *MYB34*, which play crucial roles in regulating aliphatic- and indole-glucosinolate biosynthesis, respectively, were significantly down-regulated by selenate in the young leaves and florets of broccoli [52]. Interestingly, total aliphatic GLs and total GL content in broccoli sprouts were substantially increased when the Se supplement under 1R1B+Se, 1R2B+Se, and 2R1B+Se (Figure 4). The light environment could affect GLs in brassica species. Higher percentages of blue wavelengths led to a pronounced enhancement of glucosinolate contents in pakchoi [53]. The accumulation of glucosinolates in Chinese kale sprouts shoots significantly inhibited by 30 μmol m^−2^ s^−1^ B LED (470 nm) compared with the darkness control, white LED light and R LED light treatments, while there was no obvious effect on those of the roots [54]. The canola sprouts grown under B, R, and white LEDs showed higher GL contents compared with those under B + R LEDs [55]. Therefore, Se supplementation under 1R2B LED light might be the optimal and efficient method for the production of glucosinolates.

## 4. Materials and Methods

### 4.1. Plant Materials and Cultivation Conditions

**Figure 6:**
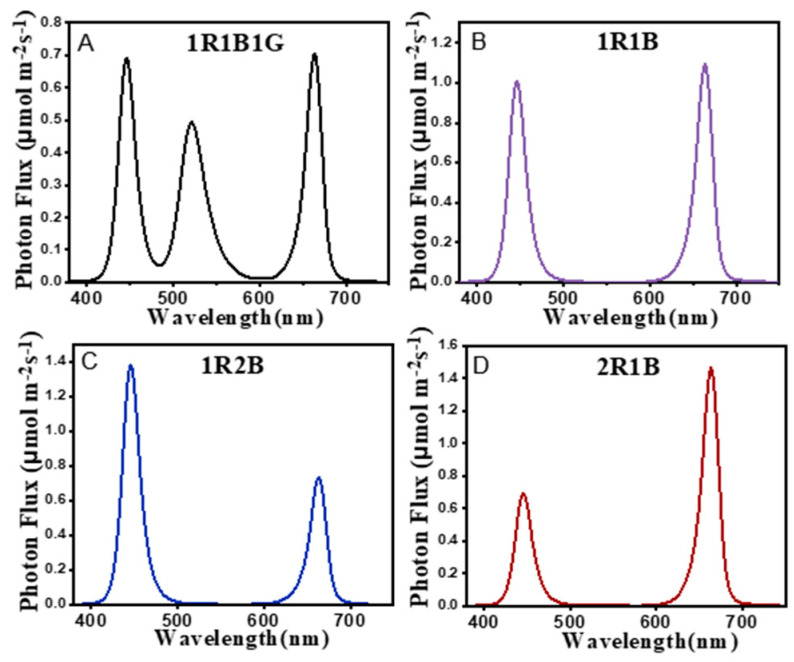
Photon flux density in the five treatments. The spectral distribution was measured by a spectrometer (ALP-01, Asensetek, Taiwan).

The study was carried out in an artificial light plant factory at South China Agricultural University. Seeds of broccoli (*Brassica oleracea* var. *italica* cv. lvhua) were obtained from SANHERB Company (Chengdu, China). Surface-sterilized seeds were soaked in 5% sodium hypochlorite for 15 min, drained and soaked with distilled water at 30 °C for 5 h, germinated and grown in plastic trays (32.5 × 24 × 4.5 cm) in the growth chamber for 4 days in dark at 25 °C with 75% relative humidity. The broccoli sprouts were transferred into a growth chamber equipped with blue (B)/red (R)/green (G) LED light. One liter of 100 μmol L^−1^ sodium selenite solution or deionized water was added to the containers, and then 10 mL of 100 μmol L^−1^ sodium selenite solution was sprayed twice per day; the control sprouts were sprayed with deionized water. There were five treatments: CK:1R1B1G LED (red 660 ± 10 nm:blue 460 ± 10 nm:green 520 ± 10 nm = 1:1:1), 1R1B1G+Se (100 μmol L^−1^ Na_2_SeO_3_), 1R1B+Se (red:blue = 1:1), 1R2B+Se (red:blue = 1:2), 2R1B+Se (red:blue = 2:1) (Figure 6 and Supplementary Table S1). The environmental conditions in this experiment were as follows: 60 μmol m^−2^ s^−1^ PPFD, 12 h/12 h (light/dark), 25 ± 1 °C, and relative humidity (RH) of 70 ± 10%, 500 ± 100 μmol·mol^−1^ CO_2_. On the seventh day, the sprouts were sampled. The sampled sprouts were frozen in liquid nitrogen, freeze-dried, milled, and stored at −40 °C for further analysis. For each treatment, three replicates were taken for analysis.

### 4.2. Biometric Measurements

Broccoli sprouts’ hypocotyl length (HL) was measured using a ruler. Fresh sprouts were weighed to obtain the fresh weight (FW), then dried at 75 °C for 72 h to obtain the dry weight (DW). Thirty broccoli sprouts were used as a sample group for each measurement.

### 4.3. Pigment Content Determination

Fresh broccoli sprouts samples (0.2 g) were dipped in 8 mL acetone ethanol mixture (acetone:ethanol = 1:1, *v*:*v*) until they turned white at ambient temperature for 24 h in darkness. The absorbance of the extract solution was measured by UV-spectrophotometer (Shimadzu UV-16A, Shimadzu, Corporation, Kyoto, Japan) at 645 nm (A_645_), 663 nm (A_663_) and 440 nm (A_440_). Chlorophyll and carotenoid concentrations were calculated as follows [56]:

Chlorophyll a (mg·L^−1^) = 12.7 ×A_663_ − 2.69 × A_645_
Chlorophyll b (mg·L^−1^) = 22.9 × A_645_ − 4.86 × A_663_
Total Chlorophyll (mg·L^−1^) = 8.02 × A_663_ + 20.20 × A_645_
Carotenoids (mg·L^−1^) = 4.7 × A_440_ − 0.27 × Total Chlorophyll
Photosynthetic pigment (mg·g^−1^) = Photosynthetic pigment (mg·L^−1^) × 8 mL × 10^−3^/0.2 g

Total anthocyanin content (TA) was measured according to the method described by Xu [57]. A ground broccoli sprout sample (1.0 g) extracted using 20 mL 60% ethanol was heated in a boiling water bath for 2 h. The homogenate was filtered into a volumetric flask. The extract solution was determined by UV-spectrophotometer at 535 nm.

### 4.4. Phytochemical Determination

Soluble sugar (SS) content was performed using the anthronesulfuric acid colorimetry method [58]. 0.5 g fresh sprouts samples were heated in a boiling water bath with 10 mL distilled water for 30 min. 0.1 mL supernatant was mixed with 1.9 mL distilled water, 0.5 mL anthrone ethyl acetate and 5 mL sulfuric acid. After shaking, the soluble sugar was detected by UV-spectrophotometer at 630 nm.

Total soluble protein (SP) contents in broccoli sprouts were determined according to the Coomassie brilliant blue G-250 dye method [59]. 0.5 g fresh samples were homogenized in 8 mL of distilled water. The homogenate was centrifuged at 3000× *g* for 10 min at 4 °C, and 0.2 mL supernatant was combined with 0.8 mL distilled water and 5 mL Coomassie brilliant blue G-250 solution (0.1 g·L^−1^). After 5 min, the soluble protein content was detected at 595 nm by UV-spectrophotometer.

Vitamin C (Vc) was assayed by the molybdenum blue spectrophotometry [60]. 0.5 g fresh samples ground into pulp with 25 mL 1% oxalic acid EDTA solution (*w*/*v*). 10 mL extracted solution was mixed with 1 mL phosphate-acetic acid, 2 mL 5% vitriol and 4 mL ammonium molybdate. After 15 min, the mixed solution was determined at 705 nm by UV-spectrophotometer.

Total phenolic compound content (TPC) was determined by the method of Vernon [61] with minor modifications. After being extracted with 8 mL straight ethanol, the homogenates centrifuged at 3000× *g* for 10 min at 4 °C. 1 mL supernatant were mixed with 0.5 mL foline-phenol and 11.5 mL 26.7% sodium carbonate for 2 h in darkness. Total phenolic content was determined by reading the absorbance at 765 nm.

The samples for total flavonoid contents (TF) assay were extracted as the method for total phenolics. The total flavonoid contents were determined by the aluminum nitrate method [62]. 1 mL extract solution was added to 11.5 mL 30% ethanol and 0.7 mL 5% NaNO_2_. After 5 min, the reaction solution was mixed with 0.7 mL 10% Al(NO_3_)_3_, and 6 min later, the mixture was added to 5 mL 5% NaOH. 10 min later, absorbance was read at 760 nm by UV-spectrophotometer.

Glucosinolates (GLs) were extracted and analyzed as previously described with slight modifications [54]. The freeze-dried samples (0.3 g) were extracted with 4 mL 70% methanol for 20 min in a 75 °C bath, which was shaken every 10 min using a vortex stirrer. 2 mL of 0.4 mol L^−1^ barium acetate was then added, and the mixture was centrifuged at 4000× *g* for 10 min (ambient temperature). The extraction was repeated twice more. Then, the supernates were combined and loaded onto a mini column containing 500 μL of DEAE-Sephadex A-25 that had been conditioned with 2 mol L^−1^ acetic acid and washed with 6 mol L^−1^ imidazole formate. After loading, the column was washed with 0.02 M sodium acetate buffer. 500 μL of a sulfatase solution (Sigma-Aldrich, Steinheim, Germany) was added, and the preparation was incubated overnight. The latter was dissolved in 2 mL of distilled water and filtered through a 0.22 μm membrane filter. HPLC analysis of desulphoglucosinolates was carried out by a Waters e2695 liquid chromatograph (Waters Crop, Milliford, MA, USA) equipped with a model 2489 UV/Vis detector and controlled by Waters Empower3 software. Samples (20 μL) were separated at 30 °C on a reversed-phase C18 column (5 μm, 250 mm × 4.6 mm; Waters SunFire C18, Waters, Milford, MA, USA). The mobile phase consisted of acetonitrile (A) and distilled water (B) and flow rate was 1.0 mL·min^−1^. Chromatograms were recorded at 229 nm. The glucosinolates were identified by their retention times and spectral data as compared by standards.

Dried broccoli sprouts were weighed and ground to powder. The content of nitrogen (N) in sprouts was determined by Ojeda [63]. Phosphorus (P) was analyzed by a vanadate–molybdate method using a spectrophotometer [64], and potassium (K) was analyzed using a flame photometer after sample digestion [65], while the content of calcium (Ca), magnesium (Mg), sulfur (S), zinc (Zn) and iron (Fe) in sprouts was determined using the atomic absorption spectrophotometry method [66]. Organic Se content was assayed using Sun’s method [67].

### 4.5. Statistics

Data were analyzed by a one-way analysis of variance (ANOVA), using SPSS 22.0 software (SPSS Inc., Chicago, IL, USA). Means comparison was performed using Duncan’s test at *p* < 0.05. Multivariate principal component analysis (PCA) was performed by and XLStat 2019 software (Addinsoft, New York, NY, USA). A heat map was generated using TBtools (http://cj-chen.github.io/TBtools/).

## 5. Conclusions

The combination of selenium and different ratios of blue/red/green narrow-band LED light quality was able to significantly increase the nutritional quality and health-promoting compounds of broccoli sprouts, including carotenoids, soluble sugars, soluble proteins, Vc, TA, TPC, TF, GLs and organic Se, in general without negatively affecting the fresh weight of sprouts. Moreover, PCA and cluster heat map showed that broccoli sprouts with 1R2B+Se treatment had higher nutritional quality and content of health-promoting compounds than other treatments. The combination of Se and 1R2B (1R2B+Se) might be beneficial to the production of selenium-biofortified high-quality broccoli sprouts.

## Figures and Tables

**Table 1:** Growth and fresh and dry weights of broccoli sprouts grown under a combination of selenium and LED light quality treatments.

Treatments	Hypocotyl Length (cm)	Fresh Weight (mg/plant)	Dry Weight (mg/plant)	Moisture Content (%)
1R1B1G	4.27 ± 0.06 a	51.44 ± 1.17 a	3.43 ± 0.07 b	93.39% ± 0.55 a
1R1B1G+Se	3.76 ± 0.05 b	49.22 ± 1.05 a	3.63 ± 0.09 ab	92.62% ± 0.40 b
1R1B+Se	3.54 ± 0.04 c	49.22 ± 0.80 a	3.61 ± 0.04 ab	92.26% ± 0.61 b
1R2B+Se	3.32 ± 0.06 d	50.78 ± 0.85 a	3.87 ± 0.17 a	92.65% ± 1.12 b
2R1B+Se	3.37 ± 0.06 d	49.22 ± 1.21 a	3.64 ± 0.07 ab	92.52% ± 0.63 b

All values in the table are expressed as mean ± standard error (*n* = 30). Different letters in the same row indicate significant differences between treatments by Duncan’s multiple range test (*p* < 0.05).

**Table 2:** Pigment contents of broccoli sprouts grown under a combination of selenium and LED light quality treatments.

Treatments	1R1B1G	1R1B1G+Se	1R1B+Se	1R2B+Se	2R1B+Se
Chl a (mg/g FW)	1.45 ± 0.10 a	1.23 ± 0.03 c	1.24 ± 0.03 c	1.43 ± 0.06 ab	1.26 ± 0.02 bc
Chl b (mg/g FW)	0.53 ± 0.05 a	0.43 ± 0.01 c	0.44 ± 0.02 bc	0.52 ± 0.02 ab	0.44 ± 0.01 bc
Chl (mg/g FW)	2.00 ± 0.14 a	1.68 ± 0.04 c	1.70 ± 0.05 c	1.96 ± 0.08 ab	1.72 ± 0.03 bc
Car (mg/g FW)	0.19 ± 0.01 b	0.22 ± 0.00 a	0.23 ± 0.00 a	0.20 ± 0.01 b	0.23 ± 0.00 a
TA (μg/g FW)	7.29 ± 0.17 b	9.23 ± 0.39 a	9.13 ± 0.24 a	9.85 ± 0.90 a	9.88 ± 0.46 a
Chl a/b	2.72 ± 0.07 a	2.87 ± 0.03 a	2.84 ± 0.03 a	2.75 ± 0.03 a	2.84 ± 0.05 a
Chl/Car	10.64 ± 1.39 a	7.47 ± 0.12 b	7.57 ± 0.29 b	9.80 ± 0.71 ab	7.53 ± 0.19 b

Chl a, Chl b, Chl, Car, and TA represent chlorophyll a, chlorophyll b, chlorophyll, carotenoid and total anthocyanin, respectively. Chl a/b represents the ratio between chlorophyll a and chlorophyll b. Chl/Car represents the ratio between total chlorophyll and carotenoids. All values in the table are expressed as mean ± standard error (*n* = 3). Different letters in the same row indicate significant differences between treatments by Duncan’s multiple range test (*p* ≤ 0.05).

**Table 3:** The content of mineral elements of broccoli sprouts grown under a combination of selenium and LED light quality treatments.

Treatment	1R1B1G	1R1B1G+Se	1R1B+Se	1R2B+Se	2R1B+Se
Macro-nutrients (g/kg DW)					
N	63.81 ± 0.32 a	62.42 ± 0.85 a	62.98 ± 0.90 a	63.17 ± 0.96 a	61.83 ± 0.95 a
P	9.55 ± 0.05 a	9.34 ± 0.21 a	9.08 ± 0.20 a	9.05 ± 0.16 a	9.05 ± 0.12 a
K	8.22 ± 0.09 a	7.38 ± 0.31 b	7.33 ± 0.06 b	7.98 ± 0.04 a	6.94 ± 0.14 b
Ca	11.70 ± 0.45 a	10.39 ± 0.06 b	10.32 ± 0.24 b	10.08 ± 0.26 b	9.8 ± 0.07 b
Mg	3.54 ± 0.07 a	3.30 ± 0.05 b	3.35 ± 0.10 ab	3.31 ± 0.06 b	3.16 ± 0.01 b
S	21.84 ± 0.58 bc	22.98 ± 0.41 ab	23.62 ± 0.23 a	21.30 ± 0.16 c	22.20 ± 0.17 bc
Micro-nutrients (mg/kg DW)					
Zn	130.09 ± 0.46 a	86.15 ± 1.70 b	86.85 ± 0.75 b	85.90 ± 1.29 b	84.04 ± 1.39 b
Fe	107.94 ± 4.96 a	108.30 ± 7.12 a	109.88 ± 5.53 a	113.38 ± 3.60 a	114.34 ± 4.68 a
Organic Se	4.48 ± 0.69 c	88.82 ± 0.30 b	107.10 ± 5.48 a	110.33 ± 4.40 a	95.86 ± 0.72 b

All values in the table are expressed as mean ± standard error (*n* = 3). Different letters in the same row indicate significant differences between treatments by Duncan’s multiple range test (*p* ≤ 0.05).

## Supplementary Materials

The following are available online, Supplementary Table S1 Details of treatments. Supplementary Figure S1 The phenotype of broccoli sprouts under combination of selenium and LED light quality treatments.
