# Supplementary material for: The Combination of Selenium and LED Light Quality Affects Growth and Nutritional Properties of Broccoli Sprouts
Source: Molecules. 2020 Oct 19;25(20):4788. doi: 10.3390/molecules25204788 (PMC7587582; doi:10.3390/molecules25204788)
Supplement: Supplementary file 1 [file molecules-25-04788-s001.pdf]

Supplementary Materials

Supplementary Table S1 Details of treatments: The light quality and concentration of sodium selenite (Na_2SeO_3) solution for this experiment. The photosynthetic photon flux density (PPFD) was maintained at 60 μmol $m^{-2}s^{-1}$.

Treatment	Red/Blue/Green LED ratios	Selenium Supplement
1R1B1G	Red: Blue: Green=1:1:1	\
1R1B1G+Se	Red: Blue: Green=1:1:1	100 μ mol L-1 Na_2SeO_3
1R1B+Se	Red: Blue=1:1	100 μ mol L-1 Na_2SeO_3
1R2B+Se	Red: Blue=1:2	100 μ mol L-1 Na_2SeO_3
2R1B+Se	Red: Blue=2:1	100 μ mol L-1 Na_2SeO_3

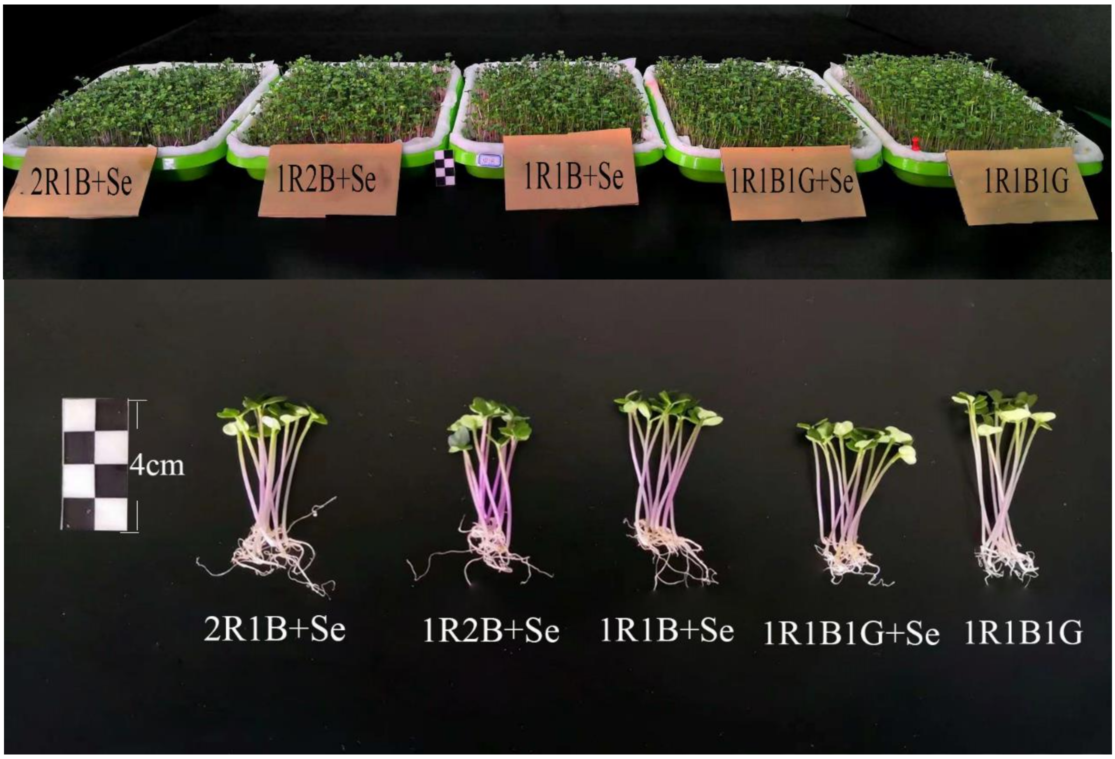

Supplementary Figure S1 The phenotype of broccoli sprouts under a combination of selenium and LED light quality treatments.
